# Prevalence of Panton-Valentine leucocidin ( *pvl*) and exfoliative toxin A ( *eta*) gene within methicillin resistant and susceptible *Staphylococcus aureus* in an urban tertiary hospital: A molecular epidemiology pilot study

**DOI:** 10.12688/f1000research.134641.3

**Published:** 2024-02-22

**Authors:** Sri Amelia, R. Lia Kusumawati, Mirzan Hasibuan, Lavarina Winda, Ridwan Balatif, Alvin Ivander

**Affiliations:** 1 Department of Microbiology, Universitas Sumatera Utara, Medan, North Sumatra, Indonesia; 2 Microbiology Laboratory, Universitas Sumatera Utara, Medan, North Sumatra, Indonesia; 3 University Hospital, Universitas Sumatera Utara, Medan, North Sumatra, Indonesia; 4 Central Laboratory, Universitas Sumatera Utara, Medan, North Sumatra, Indonesia; 5 Faculty of Medicine, Universitas Sumatera Utara, Medan, North Sumatra, Indonesia

**Keywords:** Staphylococcus aureus, Virulence factor, pvl, eta

## Abstract

**Background:**

*Staphylococcus aureus* is well known to cause a multitude of clinical manifestations, from mild to severe bloodstream infections that could lead to death. Infections are common, either in community-acquired or hospital-acquired settings, and treatment remains a challenge due to methicillin-resistant *Staphylococcus aureus* (MRSA). The pathogenesis of *S. aureus* is mediated by several cell-surface and secreted virulence factors. The virulence factors discussed in this study are Panton-Valentine leucocidin ( *pvl*) and exfoliative toxin A ( *eta*). Identifying both *pvl* and *eta* gene may help in studying bacterial pathogenesis and biology thus creating possible therapeutic pathway or intervention.

Our pilot study aimed to observe *pvl* and *eta* as virulence gene prevalence in a North Sumatera tertiary referral health center.

**Methods:**

Our study was a descriptive-analytical observational study with a cross-sectional design in which we collected isolates over a single time period. The frequency of genes is reported as a percentage comparison between MRSA and methicillin-susceptible *S. aureus* (MSSA). Qualitative gene prevalence analysis was carried out using the polymerase chain reaction (PCR).

**Results:**

Our results showed that from 38 MRSA sample isolates, 32 samples were found to be *pvl*-positive, or 84,3% of the total samples. From 40 MSSA sample isolates, one sample was found to be *pvl*-positive MSSA, or 97,5%. Regarding *eta*, from 38 MRSA sample isolates, 81,6% of the total sample did not have *eta*, while from 40 MSSA sample isolates, all samples were found to be positive for *eta*. We found that both *pvl* and *eta* were significantly more likely to be expressed in the MSSA strain.

**Conclusions:**

Our study shows that *pvl* and *eta* are more likely expressed in MSSA strains than in MRSA strains in Indonesia.

## Introduction

*Staphylococcus aureus* are Gram-positive spherical bacteria, usually arranged in a grape-like manner. This bacterium is well known to cause a multitude of clinical manifestations, from mild to severe bloodstream infections that could lead to death. Infections are common, either in community-acquired or hospital-acquired settings, and treatment remains a challenge due to multi-drug-resistant strains such as methicillin-resistant *Staphylococcus aureus* (MRSA) *.* ^1^ *S. aureus*, including MRSA, is commonly found on the skin and mucous membranes as part of the normal flora of human bodies. ^2^ While usually known as a commensal bacterium, research has shown that *S. aureus* infection is one of the most prevalent in the world. In the industrialized world, Tong *et al.*’s review showed a 10 to 30 per 100,000 person-year incidence of bacteremia caused by *S. aureus.* While overall rates may have stabilized due to the rise of antibiotics, the contribution of MRSA has fluctuated. ^3^ However, while it is well observed that staphylococcal infections caused by MRSA remain varying, in which at a specific time and location MRSA prevalence might be higher while in other time and location MRSA prevalence could be lower than predicted, studies have shown an increasing amount of MRSA infections in each decade. Hasanpour *et al.*’s research showed that before 2000, only 441 elderlies were infected with MRSA; however, this number skyrocketed to 4,365 in the 2001–2010 period and 11,987 in 2011–2022. ^4^ As such, it can be said that MRSA creates a new challenge for healthcare workers and researchers to identify its pathogenesis and thus create a sound and reliable solution.

The pathogenesis of *S. aureus* is mediated by several cell-surface and secreted virulence factors. One such virulence factor is Panton-Valentine leucocidin ( *pvl*). ^5^ *pvl* is a toxin comprising two components, LukS-PV and LukF-PV. After their secretion, both components assemble into a pore-forming heptamer on neutrophil membranes, causing neutrophil lysis. There is a significant amount of research that shows the role of *pvl* in pathogenesis; however, it remains unclear what the trends are for *pvl*-positive methicillin- sensitive *S. aureus* (MSSA) or MRSA. The molecular epidemiology and burden of *pvl*-positive MSSA or MRSA are highly variable within studies, with the US dominated by *pvl*-positive MRSA, while such bacteria have been found to be rare in Australia, which has a predominance of both *pvl*-positive MRSA and MSSA. ^6^ Prudent research toward *pvl* molecular epidemiology is vital because it is well known that *pvl* is associated with invasive disease and thus could be used as a gene marker for severe infection. In industrialized countries, such epidemiological studies have led to public health measures aimed at individuals infected with the *pvl*-producing strain. ^7^

Another such virulence factor was identified as exfoliative toxin A ( *eta*). Exfoliative toxins (ETs), also known as epidermolytic toxins, are serine proteases secreted by *S. aureus* that recognize and hydrolyze desmosome proteins in the skin. ETs have been associated with the loss of keratinocytes and cell-cell adhesion, inducing peeling of the skin and blister formation. One of the principal isoforms of exotoxins implicated in human skin damage is *eta*. Recognizing *eta* prevalence could help distinguish the extent of the damage caused by *S. aureus.* ^8^ Research has shown that *eta* is a prevalent toxin. A study done by Mohseni *et al.* revealed that 76.7% of isolates were positive for *eta* in *S. aureus* isolates. ^9^ However, research is limited toward MSSA, and there is a lack of research assessing *eta* prevalence in MRSA strains.

Due to the need for molecular epidemiological study in defining public health measures and mapping molecular profiles, especially in Indonesia and possibly in Southeast Asia, our study aimed to measure *pvl* and *eta* gene molecular prevalence at the Adam Malik General Hospital, one of Indonesia’s main tertiary referral health centers. Identifying both *pvl* and *eta* gene may help in studying bacterial pathogenesis and biology thus creating possible therapeutic pathway or intervention.

## Methods

### Study design

Our study was a descriptive-analytical observational study with a cross-sectional design, in which we collected isolates from the North Sumatera Tertiary Referral Center over a single time point which was in January 2022. Our study was carried out after it had been approved by the ethical medical research committee of the Faculty of Medicine, Universitas Sumatera Utara, Medan, Indonesia through letter no. 540/KEPK/USU/2022. Isolates were collected in a blind manner. This method was used to prevent bias in clinical correlation due to our specific study to identify molecular epidemiology at a specific time point, not to explain a phenomenon caused by a toxin or virulence factor specifically. We determined the sample size needed using the following descriptive sample formula:

$$n=\frac{{\mathrm{Za}}^{2}\mathrm{PQ}}{d^{2}}$$

To obtain 95% confidence interval, Za was pre-determined with 1.96 while d was 0.1. According to Mohseni’s study, ^8^ which found P (proportion of *eta* positive sample in population) to be 0.767, we found that our study needed at least a total sample of 68 isolates to obtain statistical power. Our study included samples that were as evenly divided into MRSA and MSSA as possible.

After we obtained the minimal sample needed, we collected isolates from the Adam Malik General Hospital Microbiology Installation using cluster random sampling. All MRSA and MSSA samples within time periods were assigned a special code which then would be processed with computer randomization tool. We obtained 38 MRSA samples and 40 MSSA samples without information regarding sample clinical history to prevent bias. All samples were collected from bacteremia patient through blood collection. Colonies suspected to be *S. aureus* were cultured in mannitol salt agar (MSA) medium, while antibiotic susceptibility tests were performed with VITEK-2 system. Suspension obtained from blood agar medium was transferred to the Eppendorf and stored at -80°C. Suspension were made by combining 10 mL of aquabidest added with bacteria colony obtained from agar and then vortexed and inserted into densitometer.

MRSA or MSSA interpretation can be done with VITEK-2 system interpretation.

### DNA extraction

DNA extraction was done from bacterial cells using Presto Mini gDNA Bacteria (Geneaid). 1 × 10 ^9^ colonies of bacterial cells were inserted in a 1.5 mL sterile tube and centrifuged at 13.000 rpm for 1 minute; afterwards, the supernatant was removed. Inside the tube, 200 μL of phosphate buffer saline (including 0.8 mL/200 μL lysozyme) were vortexed. Incubation was then performed at 20°C for 5 minutes, and then 20μL of proteinase K was added and vortexed. Incubations were then performed at 70°C for 10 minutes, then the result was added to 200 μL of absolute ethanol and vortexed. GD columns were then arranged inside the collection tube, and 400 μL of buffer W1 were centrifuged at 13,000 rpm for about 30 seconds. Then, liquids were extracted, and centrifugation was re-done with 600 μL wash buffer. After 100 μL of elution buffer were added and maintained at room temperature for 3 minutes, the tubes were filled with pure DNA. Tubes were saved in a freezer at -20°C. Our protocol was in accordance with GeneAid protocol specified inside the product kit.

### Identification of *pvl* and *eta* genes by PCR

In the PCR detection of the *pvl* and *eta* genes, specific primers were used after searching through the NCBI search engine to ensure their specificity. Table 1 lists the specifications for the primers used, and Table 2 describes our temperature program and volume.

**Table 1.** The specifications for the primers.

Name	Sequence	Amplitude size	Source publication
*eta* *forward*	5′-TTTGCTTTCTTGATTTGGATTC-3′	464 bp	Mohseni *et al*., 2018 ^9^
*eta* *reverse*	5′-GATGTGTTCGGTTTGATTGAC-3′		
*pvl* *forward*	5′-ACAAGCAAAAGAATACAGCG-3′	575 bp	Hesari *et al*., 2018 ^10^
*pvl* *reverse*	5′-GTTTTTGGCTGCTTCTCTTG-3′		

**Table 2.** Thermal cycling device temperature program, and the amount and concentration of materials required for PCR.

Cycles	Steps	Temperature	Time	Materials	
*Staphylococcus aureus*				Materials	Amounts
First step: 1 cycle	Hot Start	94	5 min	Master mix PCR 2x	12.5 uL
Second step: 35 cycles *pvl*	Denaturation Annealing Extension	94 52 72	30 s 30 s 30 s	Primer forward Primer reverse Nuclease free water Template DNA	1 uL 1 uL 8.5 uL 2 uL
35 cycles *eta*	Denaturation Annealing Extension	94 53 72	30 s 30 s 30 s		
Third step: 1 cycle	Further extension	72	30 s		

Afterward, we began PCR mastermix preparation by liquefying GoTaq Green Master Mix 2×, primers (forward and reverse), nuclease-free water and DNA template. Our primer was obtained from Integrated DNA Technologies. These were then vortexed and spun down for 10 seconds, and a PCR mix was then prepared with the materials specified below ( Table 2) to get one sample. Afterward, vortexing was done to ensure suspension homogeneity. DNA templates were then added, and the thermal cycling was carried out with the parameters listed in Table 2. In summary, for most steps, 30 seconds are sufficient, while in the first step, a 5-minute hot start at 94°C would be necessary. To detect *pvl*, 35 cycles consisting of 94, 52, and 72°C of denaturation, annealing, and extension were performed for 30 seconds. *eta* followed a similar step. Our protocol was in accordance with GeneAid protocol specified inside the product kit.

### Electrophoresis of PCR product

PCR products were electrophoresed using 2% agarose gel (Merck, Germany). A mixture of 1 λ DNA loading dye and 5 λ PCR product was loaded in the gel. Electrophoresis was performed at a voltage of 80 V. Our electrophoresis method was done in accordance with Merck instruction.

### PCR Visualization and analysis

The electrophoresis results were obtained and visualized through a UV transilluminator. PCR results were defined as positive when a contrast streak was within the primer base pair or negative when the chamber was found to be empty after visualization. As the aim of the study was to identify specific virulence gene prevalences, quantitative PCR or RNA sampling were not performed. Visualization and analysis was done in accordance with Merck instruction and default software provided by electrophoresis tool.

### Statistical analysis

SPSS version 25 and Microsoft Office Excel were used to analyze the data. The frequency of genes was reported as a percentage frequency, and a descriptive comparison was made between MRSA strain gene prevalence and that of MSSA. A bivariate analysis was performed to determine the statistical difference between groups. Due to the categorical nature of our data, the Chi-square test was chosen. Significance was proven with a p-value below 0.05 in the 95% confidence interval.

## Results

Our results showed that from 38 MRSA sample isolates, 32 samples were found to be *pvl*-positive, or 84.3% of the total samples. From 40 MSSA sample isolates, one sample was found to be *pvl*-positive MSSA, or 97.5%. Regarding *eta*, from 38 MRSA sample isolates, 81.6% of the total sample did not have *eta*, while from 40 MSSA sample isolates, all samples were found to be positive for *eta*. Afterwards, we analyzed our data with the Chi-square test to determine whether significant differences existed between *pvl* and *eta* prevalence. We found that both *pvl* and *eta* were significantly more likely to be present in the MSSA strain, with p ≤ 0.05, respectively. The results can be seen in Table 3.

**Table 3.** *pvl* and *eta* prevalence between MRSA and MSSA.

Variables	Total samples (n = 78)	MRSA (n = 38)	MSSA (n = 40)	p-value
*pvl* positive	71 (91.1%)	32 (84.3%)	39 (97.5%)	0.040 *
*pvl* negative	7 (8.9%)	6 (15.7%)	1 (2.5%)	
*eta* positive	71 (91.1%)	31 (81.6%)	40 (100%)	0.004 *
*eta* negative	7 (8.9%)	7 (18.4%)	0 (0%)	

* Chi-square test.

**Figure 1.**
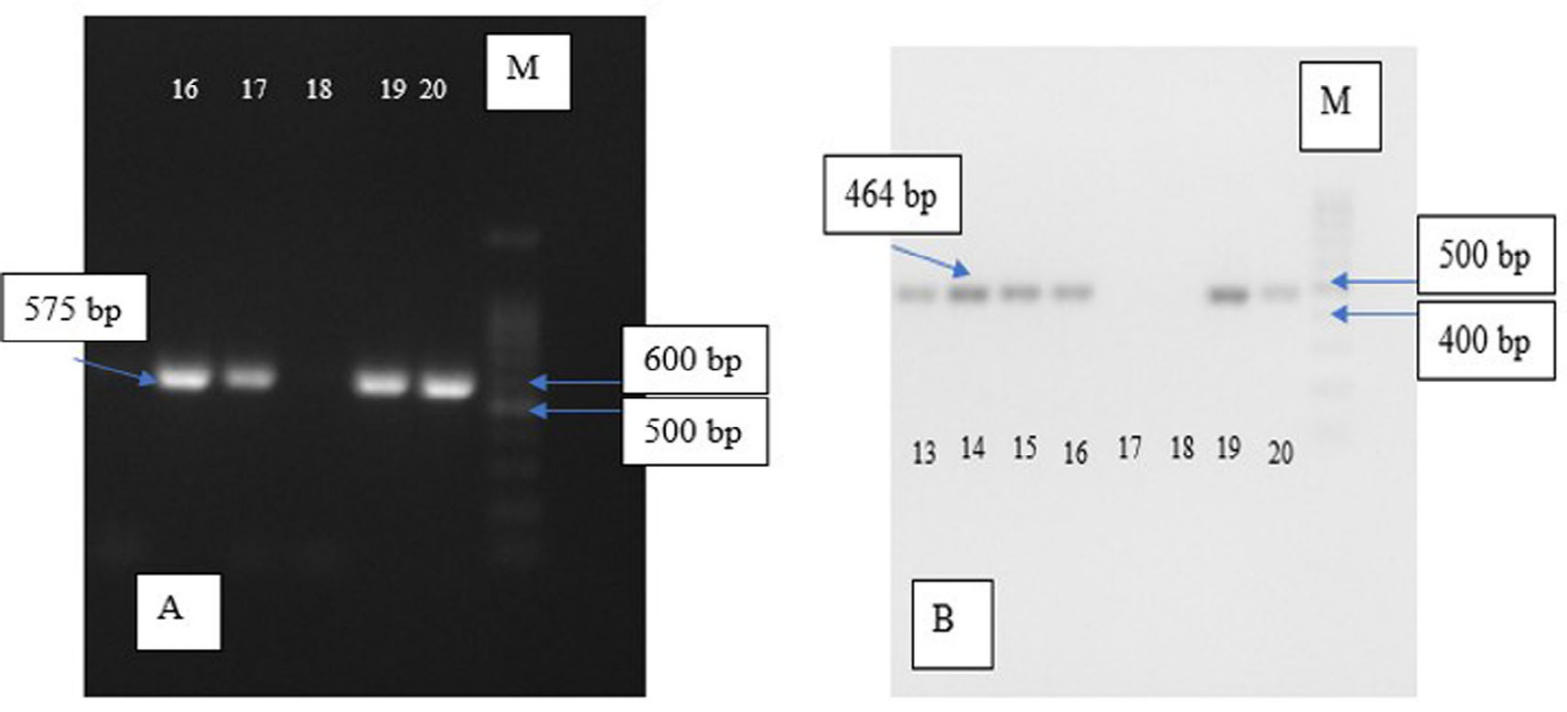
Electrophoresis of *eta* and *pvl* gene, (A) Electrophoresis of MRSA *pvl* DNA with product gene in 575 bp; chamber 18 shows negative expression while the rest was positive; (B) Electrophoresis of MRSA *eta* DNA with product gene in 464 bp; chamber 17 and 18 show absence of the target gene while the rest was positive; M = marker.

Electrophoresis visualisation for each group can be seen in Figure 1 below.

## Discussion

Our research was performed to identify the molecular epidemiology of *pvl*-positive MRSA, *pvl*-positive MSSA, and *eta* prevalence in either MRSA or MSSA, thus supporting local public health authorities in creating a sound public health measure and fulfilling data needs regarding *S. aureus* molecular epidemiology, especially in Southeast Asia. According to our results, most MRSA and MSSA were found positive for *pvl* and *eta* expression. We also found that MSSA was significantly more likely to encode both *pvl* and *eta*.

Our research shows very concerning new updates which revealed high *pvl* (+) prevalence in bacteremia patient. In a study performed by Boan *et al*. in Australia during the 2007–2009 period, *pvl*-negative and positive samples were still equally dominating, with 141 *pvl*-positive *S. aureus* and 148 *pvl*-negative strains. The Boan study did find that *PVL* didn’t significantly affect methicillin resistance, with 56% of *pvl*-positive strains found to be MRSA. However, it is interesting to note that in the Boan study, which reflects Australia’s *S. aureus* molecular epidemiology, it was shown that *pvl*-positive MRSA had begun to dominate in Western Australia. ^11^ These results concur with our study, and by reflecting its close geographical distance, our result creates a new concern that *pvl*-positive MRSA and MSSA dominate most *S. aureus* specimens.

While all of our samples were procured from bacteremia patient, it might be interesting to analyzed source of infection in *pvl* (+) isolates. Melles research in 2006 point out high percentage of *pvl* (+) *S. aureus* in soft tissue infection isolates. This was not consistent with our finding however Melles research were performed nearly two decades before and changes in bacterial biology could happen within the span of time.

Recent research performed in Gambia by Darboe *et al*. in 2019 revealed a somewhat different result at several time points, while at the same time reflecting the similar results found in our study in another time points. While it is correct to assume that the *pvl*-positive samples have increased from 2005 levels to 2015 levels, their numbers has been fluctuating at best. However, it is interesting to note that in one period of the study, the *pvl*-positive strain was as high as 90%. Darboe’s study didn’t elaborate on whether there is methicillin resistance in *pvl*-positive or *pvl*-negative strains; however, Darboe did conclude that there was no association between *pvl* and antimicrobial resistance, while emphasizing the still-low antimicrobial resistance in Gambia. ^12^ This result brought a new perspective to our study as it showed a fluctuating trend. It might be possible that some measures were taken during the lower *pvl*-positive strain infection period, and it has been found that while our results show unusually high *pvl*-positive MRSA and MSSA, this result was not irreversible.

Jaiswal’s report in 2022 revealed another different result. Their study results were not consistent with ours, whereby Jaiswal revealed only 49 out of 162 positive *pvl* strains within MRSA. ^13^ We noted that the Jaiswal study was not consistent with our study or with other studies performed in India. A study by Kaur from India reported 85.1% *pvl*-positive MRSA prevalence within the country, ^14^ similarly to D’Souza’s study in Mumbai which revealed 64% *pvl*-positive MRSA. ^15^ This doesn’t necessarily mean that the Jaiswal study was incorrect, as it emphasized the dynamic change in MRSA genetic epidemiology. In a very large country like India, even molecular epidemiology could differ between regions and provinces.

Local studies at Andalas University performed by Linosefa *et al*. revealed a more balanced number. Linosefa found only two out of 19 samples to be positive for *pvl*. ^16^ While this result was encouraging, it should be noted that this research was performed nearly a decade ago, and this research recommends the importance of surveillance, as was done in our study. This result also strengthened our hypothesis regarding how varying *pvl* prevalence could be.

Another interesting is the research done by Bhatta *et al*. in 2016 in Nepal. In his study, Bhatta observed that 90.4% of MRSA acquired in the community was *pvl*-positive, while *pvl* detected in nosocomial infections was only 7.1% positive. It was also found that *pvl*s were not associated with bloodstream infections. ^17^ While the number of samples makes it hard to draw any definitive scientific conclusion, there is a high possibility that Indonesian *S. aureus*-infected patients mostly acquire their infection from the community. Interventions toward personal hygiene or public health measures might indeed lower *pvl*-positive prevalence.

With regards to *eta*, our results also show a disturbing new discovery in which most MRSA and MSSA were positive for the *eta* gene, with MSSA being significantly more likely to have *eta*. However, this has actually been predicted by several studies. Mohseni, in his study conducted in Iraq, found that 87.3% of isolates, or 131 samples, were positive for at least ET genes, with *eta* being dominant. *eta* was found in 76.7% of the samples obtained by Mehsani. In the conclusion, Mehsani deemed the finding a serious problem as it may spread through gene transfer between strains. ^9^

A study performed by Koosha *et al*. in 2013 is, however, quite consistent with our study. In the Kooesha study, lack of both *eta* and etb genes was only detected in eight (4%) isolates, and *eta* dominated ET prevalence. ETs play a role in colonization and invasion of injured mucosa and skin. Koosha also found that the distribution of ETs was largely similar between the MRSA and MSSA groups. Finally, drug resistance was abundant in the analyzed population. ^18^ However, a study performed by Montazeri *et al*. in 2021 revealed that *eta* expression was only 23.7%. It must be emphasized that the Montazeri study was limited to cancer patients, who may have immunological disorders that could affect staphylococcal infection prevalence. ^19^ This result might also emphasize regional fluctuations in molecular epidemiology. The study from the Middle East showed an even more surprising result: none of the MRSA isolates expressed the *eta* gene, out of 76 Iraqi isolates and 49 refugee isolates. ^20^

The study conducted in the People’s Republic of China by Li *et al*. showed much more consistent results compared with our study. Li found that most MRSA and MSSA contained *eta* genes at rates of 61.8% and 55%, respectively. Li’s research compared the prevalence from year to year, and there was indeed an increasing prevalence of *eta* gene prevalence, which was 23.1% in the 2013–2014 period and increased to 80.1% in the 2018–2019 period. ^21^ Our results revealed that Li’s increasing trend is persisting even in Indonesia.

A systematic review was conducted regarding *eta*-positive *S. aureus* in Iran alone. It showed quite variable results. Fooladi’s study showed 92.7% *Staphylococcus aureus* were positive for *eta*. ^22^ On the other side of the spectrum, *eta* detection could be as low as 0%, as shown in the Rahimi study in 2018. ^23^ This systematic review showed that even between regions, the variability of the *S. aureus* virulence gene is quite large. ^24^

Lastly, a study in Indonesia done by Santosaningsih *et al*. in 2017 showed that only 11.3% of patients encoded *eta* gene. ^25^ This result shows the importance of our study due to the very variable and volatile molecular epidemiology of *S. aureus.* Modifiable factors can help reduce the burden of disease.

The limitation of our study was the blind setting. While blind research might be ideal to recognize molecular epidemiology while preventing specific clinical bias ( *e.g.*, lower *pvl* prevalence in bloodstream infection), ^17^ a clinical-to-molecular genetics relationship study could show the magnitude of this effect. Other limitations include our single-center study. However, our hospital is the main tertiary referral center in Sumatera Island, so it could reflect Staphylococcal molecular epidemiology in all of Sumatera. It still must be emphasized that most infections occur in the acute phase, and milder strains of *S. aureus* might have been treated in a primary health center. Another limitation is the use of bacteremia patient, further research should attempt to describe the prevalence of *pvl* and *eta* genes among either MSSA or MRSA clinical isolates based on the type of specimen. Through this method, severity of disease may be assessed. We proposed further research with double-blind, multi-center and clinical studies to elaborate the existing body of knowledge explained either in this study or in other studies.

## Conclusions

Our study shows that *pvl* and *eta* are more likely expressed in MSSA strains than in MRSA strains in Indonesia. This is a relatively new finding and could have significant implications for treating MSSA, as *pvl* and *eta* have been deemed virulence factors that could worsen disease progression. Public health measures are necessary, and continued surveillance is important to ensure that *pvl* or *eta* prevalence as a virulence factor decreases with time and effort.

## Data Availability

Figshare: Electrophoresis result of Staphylococcus aureus clinical isolates from Adam Malik General Hospital, https://doi.org/10.6084/m9.figshare.22796012.v1. ^26^ Figshare: Result of *ETA* and *PVL* PCR and Cefoxitime Screening of Clinical Isolates, https://doi.org/10.6084/m9.figshare.22849361.v1. ^27^ Data are available under the terms of the Creative Commons Attribution 4.0 International license (CC-BY 4.0).
