# Supplementary material for: Prevalence of Panton-Valentine leucocidin ( pvl) and exfoliative toxin A ( eta) gene within methicillin resistant and susceptible Staphylococcus aureus in an urban tertiary hospital: A molecular epidemiology pilot study
Source: F1000Res. 2024 Feb 22;12:1002. Originally published 2023 Aug 18. [Version 3] doi: 10.12688/f1000research.134641.3 (PMC10905128; doi:10.12688/f1000research.134641.3)
Supplement: Peer review history

# Reviewer report: Reviewer response for version 3

**DOI:** 10.5256/f1000research.162867.r249245

**Published:** 2024-02-24

**Authors:** Dewi Santosaningsih

**Affiliations:** 1 Department of Clinical Microbiology, Faculty of Medicine, Universitas Brawijaya, Malang, East Java, Indonesia

The authors have revised the manuscript as suggested. I have no further comments.

Is the work clearly and accurately presented and does it cite the current literature?

Partly

If applicable, is the statistical analysis and its interpretation appropriate?

Partly

Are all the source data underlying the results available to ensure full reproducibility?

Partly

Is the study design appropriate and is the work technically sound?

Partly

Are the conclusions drawn adequately supported by the results?

Partly

Are sufficient details of methods and analysis provided to allow replication by others?

Partly

Reviewer Expertise:

Antimicrobial resistance; infection control; Staphylococcus aureus

I confirm that I have read this submission and believe that I have an appropriate level of expertise to confirm that it is of an acceptable scientific standard.

# Reviewer report: Reviewer response for version 2

**DOI:** 10.5256/f1000research.160789.r237328

**Published:** 2024-02-15

**Authors:** Dewi Santosaningsih

**Affiliations:** 1 Department of Clinical Microbiology, Faculty of Medicine, Universitas Brawijaya, Malang, East Java, Indonesia

**Manuscript title:** Prevalence of Panton-Valentine leucocidin (pvl) and exfoliative toxin A (eta) gene within methicillin resistant and sensitive Staphylococcus aureus in an urban tertiary referral hospital: A molecular epidemiology pilot study

**Aim:** To observe pvl and eta as virulence gene prevalence in a North Sumatera tertiary referral health center.

   − Please use methicillin-susceptible Staphylococcus aureus instead of methicillin-sensitive Staphylococcus aureus not only in the title but also in the abstract and the entire of text. Please recheck.

   − The genes should be written in italic (e.g pvl, eta) not only in the title but also in the abstract and the entire of text. Please recheck.

**METHODS:**

   − Study design: "Sample lab numbers with MRSA and MSSA..." is not understandable. Please rephrase.

   − The authors mention oxidase test in the methods section: “The samples were then cultured on blood agar medium, and colonies suspected of being Staphylococcus aureus were tested using Gram staining, catalase, oxidase, and coagulase tests”. In order to identify staphylococci, it is not necessary to do it.

   

   − The authors described “The samples were then cultured on blood agar medium…” but then also described “Colonies suspected to be S. aureus were cultured in mannitol salt agar (MSA) medium, ….”. Please explain the media agar used for culture clearly.

   − The antibiotic susceptibility test was not clear yet. Apparently, the authors use Kirby Bauer method mainly to perform antibiotic susceptibility test. In the particular conditions, the authors used VITEK2 system. Please describe clearly.

   − The CLSI interpretation for Staphylcoccus aureus against cefoxitin: Susceptible or Resistant. No intermediate category. Please check the CLSI 2023." − PCR: did the authors include both positive and negative control? Please describe it.

   − Please mention the citation in each part of methods.

**RESULTS:**

 The authors described: The results of our PCR tests showed that from 38 MRSA sample isolates, six samples were found to be pvl-negative, or 15.7% of total samples. From 40 MSSA sample isolates, one sample was found to be pvl-negative ……. Please mention the positive results instead of negative results.

**DISCUSSION:** Due to several limitations of this study, what is the next steps? Please describe at the end of the discussion section.

Is the work clearly and accurately presented and does it cite the current literature?

Partly

If applicable, is the statistical analysis and its interpretation appropriate?

Partly

Are all the source data underlying the results available to ensure full reproducibility?

Partly

Is the study design appropriate and is the work technically sound?

Partly

Are the conclusions drawn adequately supported by the results?

Partly

Are sufficient details of methods and analysis provided to allow replication by others?

Partly

Reviewer Expertise:

Antimicrobial resistance; infection control; Staphylococcus aureus

I confirm that I have read this submission and believe that I have an appropriate level of expertise to confirm that it is of an acceptable scientific standard, however I have significant reservations, as outlined above.

# Reviewer report: Reviewer response for version 2

**DOI:** 10.5256/f1000research.160789.r237327

**Published:** 2024-02-08

**Authors:** Amin Soebandrio

**Affiliations:** 1 Clinical Microbiology, Faculty of Medicine, University of Indonesia, Depok, West Java, Indonesia

The author has responded to all my questions, comments, and suggestions. I have no further questions or comments. Thank you.

Is the work clearly and accurately presented and does it cite the current literature?

Partly

If applicable, is the statistical analysis and its interpretation appropriate?

I cannot comment. A qualified statistician is required.

Are all the source data underlying the results available to ensure full reproducibility?

Partly

Is the study design appropriate and is the work technically sound?

Yes

Are the conclusions drawn adequately supported by the results?

Yes

Are sufficient details of methods and analysis provided to allow replication by others?

Yes

Reviewer Expertise:

Clinical Microbiology, Immunology, Molecular Biology.

I confirm that I have read this submission and believe that I have an appropriate level of expertise to confirm that it is of an acceptable scientific standard.

# Reviewer report: Reviewer response for version 1

**DOI:** 10.5256/f1000research.147714.r202379

**Published:** 2023-11-24

**Authors:** Amin Soebandrio

**Affiliations:** 1 Clinical Microbiology, Faculty of Medicine, University of Indonesia, Depok, West Java, Indonesia

Methodology:

1. Please clarify the followings:
  1.  Antibiotic discs were placed inside or on MHA? 
  2. What does it mean "after 24 hours of incubation"?
  3. What suspension was transferred?  
2. DNA extraction
  1. ​Bacterial cells should be first suspended before centrifugation. In what solution or buffer the  bacteria was suspended.
  2. Buffer contained Lysozyme was then added and vortexed. What buffer?
  3. 200uL of absolute ethanol was then added and vortexed.

Sentences in this paragraph should be rewritten to improve clarity or the procedures.

 Discussion.

 The percentage of PVL gene in community-acquired and hospital-acquired MRSA and MSSA should be described and discussed. More references should be included in the discussion and conclusion development. Among others, Melles DC, van Leeuwen WB, Boelens H, Peeters JK, Verbrugh HA, van Belkum A. Panton-Valentine Leukocidin Genes in Staphylococcus aureus. Emerg Infect Dis. 2006;12(7):1174-1175. https://doi.org/10.3201/eid1207.050865.

 Others.

 Any abbreviation should be described when it appears for the first time.

 Example: ET.

Is the work clearly and accurately presented and does it cite the current literature?

Partly

If applicable, is the statistical analysis and its interpretation appropriate?

I cannot comment. A qualified statistician is required.

Are all the source data underlying the results available to ensure full reproducibility?

Partly

Is the study design appropriate and is the work technically sound?

Yes

Are the conclusions drawn adequately supported by the results?

Yes

Are sufficient details of methods and analysis provided to allow replication by others?

Yes

Reviewer Expertise:

Clinical Microbiology, Immunology, Molecular Biology.

I confirm that I have read this submission and believe that I have an appropriate level of expertise to confirm that it is of an acceptable scientific standard, however I have significant reservations, as outlined above.

## References

[1] : Panton-Valentine Leukocidin Genes inStaphylococcus aureus. *Emerging Infectious Diseases* .2006;12(7) : 10.3201/eid1207.050865 1174-1175 10.3201/eid1207.050865 PMC337573416848048

# Response

**Published:** 2023-12-29

**Authors:** Sri Amelia

**Affiliations:** Microbiology Department Faculty Medicine Universitas Sumatera Utara, Indonesia

Methodology:

1. Please clarify the followings:
  1.  Antibiotic discs were placed inside or on MHA? 
  2. What does it mean "after 24 hours of incubation"?
  3. What suspension was transferred?  

**Thank you for the correction all unclear parts in methodology has been corrected.**

1. DNA extraction
  1. ​Bacterial cells should be first suspended before centrifugation. In what solution or buffer the  bacteria was suspended.
  2. Buffer contained Lysozyme was then added and vortexed. What buffer?
  3. 200uL of absolute ethanol was then added and vortexed.

Sentences in this paragraph should be rewritten to improve clarity or the procedures.

**Thank you for the correction all unclear parts in methodology has been corrected.**

 Discussion.

 The percentage of PVL gene in community-acquired and hospital-acquired MRSA and MSSA should be described and discussed. More references should be included in the discussion and conclusion development. Among others, Melles DC, van Leeuwen WB, Boelens H, Peeters JK, Verbrugh HA, van Belkum A. Panton-Valentine Leukocidin Genes in Staphylococcus aureus. Emerg Infect Dis. 2006;12(7):1174-1175. https://doi.org/10.3201/eid1207.050865.

**We found this research interesting and we had added this research in our discussion. Thank you**

 Others.

 Any abbreviation should be described when it appears for the first time.

 Example: ET.

**Thank you for the correction. We had followed the review as best as we could.**

# Reviewer report: Reviewer response for version 1

**DOI:** 10.5256/f1000research.147714.r221584

**Published:** 2023-11-24

**Authors:** Dewi Santosaningsih

**Affiliations:** 1 Department of Clinical Microbiology, Faculty of Medicine, Universitas Brawijaya, Malang, East Java, Indonesia

Review

 Manuscript title: Prevalence of Panton-Valentine leucocidin (pvl) and exfoliative toxin A (eta) gene within methicillin resistant and sensitive *Staphylococcus aureus* in an urban tertiary referral hospital: A molecular epidemiology pilot study

 Aim: to observe pvl and eta as virulence gene prevalence in a North Sumatera tertiary referral health center.

 General comments:

 This study is important to predict the clinical manifestation of Staphylococcus aureus infection based on the properties of virulence genes including *pv*l and *eta*.

- According to the previous studies, *pvl *and *eta* genes were more likely found among *Staphylococcus aureus *isolated from community setting. In this study, the authors have evaluated the prevalence of the *pvl* and *eta* genes among clinical *Staphylococcus aureus *isolated from a tertiary care hospital in Indonesia. **The reasons or background for evaluating the prevalence of pvl and eta genes in the hospital setting have not been described yet (both in the abstract and body text).**
- The academic merit of this study have not been explained yet.
- English grammar issue should be improved.

Title:

- The *genes* should be written in *italic *(e.g *pvl*, *eta*)
- Please use methicillin- **susceptible** *Staphylococcus aureus* instead of methicillin- **sensitive** *Staphylococcus aureus*
- A tertiary care hospital in Indonesia indicates a referral hospital, therefore the title contains redundancy.

Abstract:

 The abstract was not described the study clearly, it should be re-arranged. The abstract would be better after the authors revised the body text (introduction, materials and methods, results, and discussion)

 Introduction:

 The authors should be described the **purpose** of the study clearly. The **urgency of the study** was not described in the background yet.

 Materials and methods:

 The **type of specimens** of clinical isolates *Staphylococcus aureus* collected in this study were not mentioned yet.

 The **method of identification and antibiotic susceptibility test** of the clinical isolates *Staphylococcus aureus* were not described clearly yet.

 Results:

 The description of results section should be re-arranged to be clearer and more understandable. The authors can start by explaining the prevalence of *Staphylococcus aureus* (overall) and then divide it into MRSA (%) and MSSA (%). Furthermore, the authors could describe **the prevalence of *pvl* and *eta* genes among either MSSA or MRSA clinical isolates based on the type of specimen**. How many *pvl* gene was positive among either MSSA or MRSA isolates from blood, sputum, and wound? Then, the severity of disease could be predicted.

 Discussion:

 The authors should elaborate the discussion section based on the results section, therefore the conclusion will be drawn adequately supported by the results.

Is the work clearly and accurately presented and does it cite the current literature?

Partly

If applicable, is the statistical analysis and its interpretation appropriate?

Partly

Are all the source data underlying the results available to ensure full reproducibility?

Partly

Is the study design appropriate and is the work technically sound?

Partly

Are the conclusions drawn adequately supported by the results?

Partly

Are sufficient details of methods and analysis provided to allow replication by others?

Partly

Reviewer Expertise:

Antimicrobial resistance; infection control; Staphylococcus aureus

I confirm that I have read this submission and believe that I have an appropriate level of expertise to confirm that it is of an acceptable scientific standard, however I have significant reservations, as outlined above.

# Response

**Published:** 2023-12-29

**Authors:** Sri Amelia

**Affiliations:** Microbiology Department Faculty Medicine Universitas Sumatera Utara, Indonesia

General comments:

 This study is important to predict the clinical manifestation of Staphylococcus aureus infection based on the properties of virulence genes including *pv*l and *eta*.

- According to the previous studies, *pvl *and *eta* genes were more likely found among *Staphylococcus aureus *isolated from community setting. In this study, the authors have evaluated the prevalence of the *pvl* and *eta* genes among clinical *Staphylococcus aureus *isolated from a tertiary care hospital in Indonesia. **The reasons or background for evaluating the prevalence of pvl and eta genes in the hospital setting have not been described yet (both in the abstract and body text).**
- The academic merit of this study have not been explained yet.
- English grammar issue should be improved.

**We had attempted to fix this issue, hopefully while minor, this could improve our manuscript.**

 Title:

- The *genes* should be written in *italic *(e.g *pvl*, *eta*)
- Please use methicillin- **susceptible** *Staphylococcus aureus* instead of methicillin- **sensitive** *Staphylococcus aureus*
- A tertiary care hospital in Indonesia indicates a referral hospital, therefore the title contains redundancy.

**Thank you for the correction. We had fixed the issue.**

 Abstract:

 The abstract was not described the study clearly, it should be re-arranged. The abstract would be better after the authors revised the body text (introduction, materials and methods, results, and discussion)

**Thank you for the correction. We had fixed the issue.**

 Introduction:

 The authors should be described the **purpose** of the study clearly. The **urgency of the study** was not described in the background yet.

**Thank you for the correction. We had fixed the issue.**

 Materials and methods:

 The **type of specimens** of clinical isolates *Staphylococcus aureus* collected in this study were not mentioned yet.

 The **method of identification and antibiotic susceptibility test** of the clinical isolates *Staphylococcus aureus* were not described clearly yet.

**Thank you for the correction. We had fixed the issue.**

 Results:

 The description of results section should be re-arranged to be clearer and more understandable. The authors can start by explaining the prevalence of *Staphylococcus aureus* (overall) and then divide it into MRSA (%) and MSSA (%). Furthermore, the authors could describe **the prevalence of *pvl* and *eta* genes among either MSSA or MRSA clinical isolates based on the type of specimen**. How many *pvl* gene was positive among either MSSA or MRSA isolates from blood, sputum, and wound? Then, the severity of disease could be predicted.

**We couldn't fix this issue, all samples were obtained from bacteremia patient through blood collection. No other samples were obtained through other methods. However this may be the limitation of our studies thus we had described this in limitation.**

 Discussion:

 The authors should elaborate the discussion section based on the results section, therefore the conclusion will be drawn adequately supported by the results.

**We had attempted to fix this issue.**
